# Recurrent Dacryostenosis as Initial Presentation of Sarcoidosis

**DOI:** 10.1155/2012/870527

**Published:** 2012-09-09

**Authors:** Marie Julie Blouin, Daniel O. Black, Gaetan Fradet

**Affiliations:** ^1^Enfant-Jesus Hospital, CHA, Quebec, QC, Canada G1J 1Z4; ^2^Saint-Sacrement Hospital, CHA, Quebec, QC, Canada G1S 4L8

## Abstract

Sarcoidosis is a multisystem granulomatous disease with an unknown etiology. It most commonly affects young and middle-aged females. It can affect any organ, but mostly lung, skin, and eyes. Up to half of patients are asymptomatic and the disease is often detected incidentally on abnormal chest radiography. We report the case of a 31-year-old male with bilateral recurrent dacryostenosis. The nasolacrimal obstruction was the initial manifestation of systemic sarcoidosis.

## 1. Introduction

Sarcoidosis is a multisystem granulomatous inflammation of unknown etiology [1]. It typically affects young adults and is slightly more common in women than men [2]. It is often detected incidentally by radiographic chest abnormalities on a routine exam prior to the development of symptoms [3]. It is characterized pathologically by the presence of noncaseating granulomas in affected organs [4]. The epithelioid cells are found histologically in the affected tissue wich form granulomas, secrete cytokines, and angiotensin converting enzyme (ACE) [5]. Although uveitis is the most common ocular manifestation, any part of the eye, orbit, and lacrimal system may be involved [6]. Literature review reveals rare reports of nasolacrimal obstruction. Approximately 10 to 15% of patients with sarcoidosis exhibit otorhinolaryngologic manifestations, but these are rarely the initial presentation [7].

## 2. Case Presentation

This is a 31-year-old male diagnosed with nasolacrimal sarcoidosis. He first presented complaining of bilateral epiphora worse on the left. There is no history of endonasal surgery nor facial trauma. He complained of a chronic nasal obstruction and had been treated for sinusitis in the past. He was otherwise asymptomatic and in good general health.

**Figure 1:**
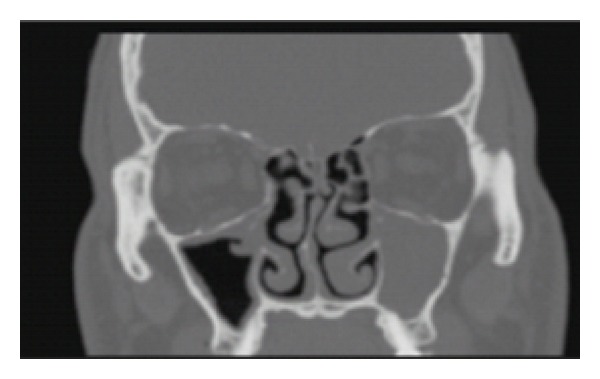
Computed tomography of the paranasal sinuses showing complete obstruction of left maxillary sinus and signs of chronic maxillary sinusitis on right side.

Exam revealed complete dacryostenosis of the left and partial obstruction of the right ducts. He was initially presumed to have idiopathic obstruction and underwent external left dacryocystorhinostomy. On followup one year later, he presented with complete obstruction on the right and bilateral chronic maxillary sinusitis as seen on sinus CT (Figure 1). We performed endoscopic dacryocystorhinostomy on the right, right anterior ethmoidectomy, and bilateral maxillary sinusotomy.

**Figure 2:**
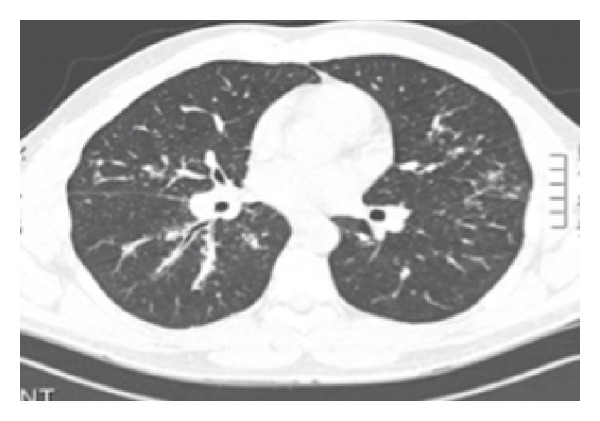
Pulmonary computed tomography showing bilateral hilar adenopathy and parenchymatous infiltration.

Histologic evaluation of ethmoidal tissues revealed granulomatous inflammation compatible with sarcoidosis. Pulmonary CT demonstrated bilateral hilar adenopathy and parenchymatous infiltration consistent with stage 2 sarcoidosis (Figure 2). Angiotensin converting enzyme was 45 (upper normal limit). Oral corticotherapy was given for 6 weeks to reduce risk of recurrent nasolacrimal obstruction.

**Figure 3:**
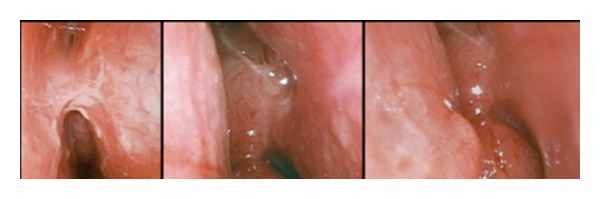
Endoscopic photograph showing right endonasal synechia. On last followup, 2 years postoperatively, lacrimal drainage remained patent without recurrence of synechia. He was followed in ophthalmology for anterior uveitis, but was otherwise asymptomatic.

On follow-up exam one year later, we noted right endonasal synechia (Figure 3). The patient subsequently underwent a third surgery during which removal of the synechia, bilateral turbinectomy, septoplasty for correction of left septal deviation, and left maxillary sinusotomy was performed. Tissue obtained intraoperatively once again revealed granulomas compatible with sarcoidosis. 

## 3. Discussion

Sarcoidosis affects primarily the respiratory tract. Although periorbital involvement is uncommon, any part of of the eye, orbit, and adnexal structures may be involved. The lacrimal gland seem to be the most affected, followed by the orbit, eyelids and the lacrimal sac [8]. There are relatively few literature reports describing nasolacrimal and adnexal sarcoidosis. 

Demirci and Christianson [8] report a series of 30 patients with orbital and adnexal involvement, 2 of whom were found to have lacrimal sac sarcoidosis. Between 1991 and 2001, Anderson et al. retrospectively reviewed a series of 377 specimens obtained during dacryocystorhinostomy. 8 specimens tested from 5 different patients revealed sarcoidosis. All 5 patients had a preoperative diagnosis systemic disease [9]. 

Although there is some nasolacrimal involvement cases reported in literature, most of them presented with known sarcoidosis. Fergie et al. reported 4 patients with nasolacrimal involvement, 3 of whom did not show evidence of systemic disease [10]. Vasquez et al. cited 16 cases in the literature with sarcoidosis of the lacrimal pathways (1938 to 1985). 15 of them showed clear evidence of systemic disease [11]. These reports indicate that nasolacrimal involvement may develop, but rarely occurs in isolation. 

Moreover, the utility of routine histopathologic examination of tissue obtained during DCR is controversial. While most nasolacrimal obstructions are idiopathic, some authors recommend histopathologic analysis in young patients, as we report here, and in the context of atypical clinical presentation. Heindl et al. performed biopsy of the lacrimal sac in 19 of 500 cases which were found to be atypical. 3 out of 19 biopsies performed were positive for sarcoidosis [12]. 

Tucker et al. obtained 162 lacrimal sac biopsy specimens in 150 consecutive patients undergoing dacryocystorhinostomy for clinical primary-acquired nasolacrimal duct obstruction [13]. Biopsies only revealed sarcoid granuloma in one case.

## 4. Conclusion

Nasolacrimal obstruction as an initial presentation of sarcoidosis is rare. Although uncommon and unrelated to specific symptoms, this condition should be considered in the differential diagnosis of epiphora and sinus complaints. This case emphasizes the clinical purpose of routine histopathological analysis of the lacrimal sac when performing DCR.
